# Supplementary material for: Identification of photoperiod-induced specific miRNAs in the adrenal glands of Sunite sheep (Ovis aries)
Source: Front Vet Sci. 2022 Jul 22;9:888207. doi: 10.3389/fvets.2022.888207 (PMC9354845; doi:10.3389/fvets.2022.888207)
Supplement: Supplementary file 1 [file Data_Sheet_1.ZIP › Supplementary file/miRDeep2 file.zip!/miRDeep2 file/Know_Structure/LP42A1/oar-mir-25.pdf]

miRBase precursor	: oar-mir-25
Total read count	: 19859
oar-miR-25 read count	: 19760
remaining reads	: 99

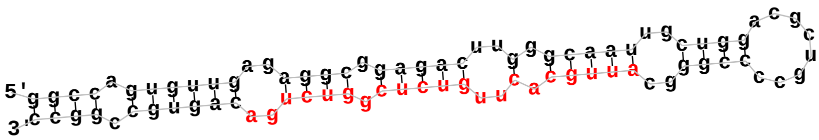

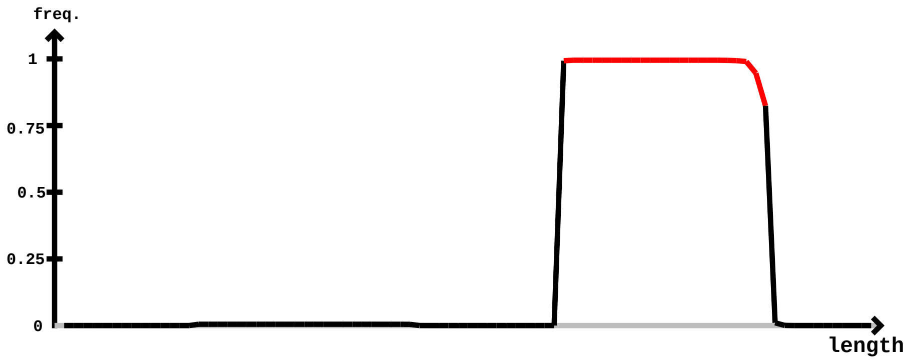

oar-miR-25

```
5'- ggccaguguugagaggcggagacuugggcaauugcuggacgcugccccgggcauugcacuugucucggucugacagugccggcc   -3'    exp
    ((((.((((((..((((.(((((..(.(((((..((((........))))..))))).)..))))).))))..)))))).))))    reads      mm        sample
    .............aggcggagacuugggcaau....................................................    1          0         seq
    .............aggcggagacuugggcaauug..................................................    1          0         seq
    .............aggcggagacuugggcaauugc.................................................    6          0         seq
    .............aggcggagacuugggcaauugcu................................................    90         0         seq
    .............aggcggagacuugggcaauugcug...............................................    1          0         seq
    ..................................................gcauugcacuugucucggucu.............    1          0         seq
    ...................................................cauugcacuugucucgg................    11         0         seq
    ...................................................cauugcacuugucucggu...............    23         0         seq
    ...................................................cauugcacuugucucgguc..............    65         0         seq
    ...................................................cauugcacuugucucggucu.............    868        0         seq
    ...................................................cauugcacuugucucggucug............    2420       0         seq
    ...................................................cauugcacuugucucggucuga...........    16126      0         seq
    ...................................................cauugcacuugucucggucugac..........    195        0         seq
    ...................................................cauugcacuugucucggucugaca.........    12         0         seq
    ....................................................auugcacuugucucggu...............    1          0         seq
    ....................................................auugcacuugucucggucu.............    3          0         seq
    ....................................................auugcacuugucucggucug............    5          0         seq
    ....................................................auugcacuugucucggucuga...........    29         0         seq
    .......................................................gcaUuugucucggucuga...........    1          1         seq
```
